# Chronic intermittent hypoxia exacerbates hepatic steatosis in a microbiota-dependent manner in lean mice

**DOI:** 10.1128/msystems.00163-26

**Published:** 2026-05-04

**Authors:** Xiaoman Zhang, Anyuan Zhong, Yupu Liu, Jianyin Zou, Meizhen Gu, Xiaoyue Zhu, Huajun Xu, Shankai Yin

**Affiliations:** 1 Department of Otolaryngology Head and Neck Surgery and Otolaryngology, Institute of Shanghai Jiao Tong University, Shanghai Sixth People's Hospital Affiliated to Shanghai Jiao Tong University School of Medicine, Shanghai, China; 2 Department of Respiratory and Critical Care Medicine, The Second Affiliated Hospital of Soochow University, Suzhou, Jiangsu, China; 3 Department of Otolaryngology-Head and Neck Surgery, Shanghai Children's Hospital, School of Medicine, Shanghai Jiao Tong University 12474, Shanghai, China; Pacific Northwest National Laboratory, Richland, Washington, USA

**Keywords:** obstructive sleep apnea, intermittent hypoxia, hepatic steatosis, inflammation

## Abstract

Chronic intermittent hypoxia (CIH), a hallmark pathological feature of obstructive sleep apnea (OSA), is extensively linked to hepatic steatosis in high-fat-diet-induced mice. However, the association between CIH and hepatic steatosis in lean mice, as well as the potential involvement of gut microbiota-related mechanisms, remains poorly understood. Four hundred participants in the Shanghai Sleep Health Study were included to assess the association between apnea-hypopnea index (AHI) and hepatic steatosis index (HSI). To characterize CIH-associated phenotypes and explore microbiota-related alterations in lean mice, liver histology, inflammatory cytokine profiling, metagenomic sequencing with antibiotic intervention, plasma untargeted metabolomics, and liver transcriptomics were performed. As a result, AHI was positively associated with HSI in non-obese participants. In lean mice, 16-week CIH alone induced hepatic steatosis and inflammation, accompanied by significant alterations in gut microbiota composition. Antibiotic treatment attenuated hepatic steatosis and inflammation in 16-week CIH-exposed mice. Metagenomic analysis revealed CIH-associated depletion of *Bacteroides uniformis*, which was reversed by antibiotic treatment. Plasma metabolomic profiling identified deoxycholic acid as a metabolite exhibiting opposite, phenotype-aligned alterations between CIH and CIH plus antibiotic groups and showing the strongest correlation with *Bacteroides uniformis* abundance. In parallel, liver transcriptomics revealed coordinated alterations in bile acid-related metabolic pathways and PPAR signaling consistent with CIH-induced and antibiotic-sensitive metabolic remodeling. Together, these findings indicate that prolonged CIH exposure induces hepatic lipid accumulation in lean mice and is associated with coordinated, antibiotic-sensitive alterations in gut microbiota composition, bile acid metabolism, and hepatic transcriptional programs, suggesting a potential involvement of gut microbiota-bile acid-liver interactions in CIH-associated hepatic steatosis.

**IMPORTANCE:**

Obstructive sleep apnea (OSA) is increasingly recognized as a contributor to metabolic dysfunction, yet its role in hepatic steatosis independent of obesity remains incompletely understood. This study shows that chronic intermittent hypoxia (CIH), a defining pathological feature of OSA, is sufficient to induce hepatic steatosis and inflammation in lean mice, independent of dietary manipulation. These findings broaden current understanding of OSA-associated liver disease beyond the context of obesity and metabolic syndrome. By integrating metagenomic sequencing, plasma metabolomics, and liver transcriptomics, this work highlights coordinated alterations in gut microbial composition, bile acid profiles, and hepatic lipid-related transcriptional programs associated with CIH exposure. Depletion of *Bacteroides uniformis* and elevation of deoxycholic acid were linked to CIH-induced hepatic phenotypes and were sensitive to antibiotic intervention, supporting a contributory role of gut microbiota-bile acid interactions in this process. Together, these findings underscore the potential importance of gut microbiota-host metabolic crosstalk in OSA-associated hepatic steatosis and suggest that microbiota- or bile acid-targeted strategies may warrant further investigation as adjunctive approaches for risk stratification and therapeutic intervention in OSA-related liver disease.

## INTRODUCTION

Obstructive sleep apnea (OSA) is one of the most common sleep-disordered breathings characterized by frequent airway collapse or obstruction, resulting in intermittent hypoxia (IH) and sleep fragmentation (1). OSA is associated with multiple physiological consequences, including cognitive and metabolic dysfunctions (2, 3). Acute IH, involving short-duration and mild hypoxia exposures, has been employed for therapeutic purposes (4), whereas chronic IH (CIH), consisting of prolonged and severe exposures (usually 8 h/day for several weeks), is commonly used to simulate OSA conditions. CIH has been implicated in the pathogenesis of a wide variety of chronic disorders, including endothelial dysfunction and cognitive impairment (5, 6). More and more evidence has indicated that sympathetic nervous overactivity, oxidative stress, inflammation, dysregulated HIF-1α related responses, and mitochondrial dysfunction might contribute to CIH-induced injury (7–11).

CIH has been identified as a risk factor that exacerbates liver injury and promotes the progression of non-alcoholic fatty liver disease (NAFLD) and non-alcoholic steatohepatitis (NASH) (12). Xiong et al. have revealed that CIH triggers selective autophagy by activating HIF-1α, leading to the degradation of DNA repair enzyme and exacerbating DNA damage in hepatocytes, thereby worsening the pathogenesis of NASH (13). Moreover, HIF-1α and IH independently worsen liver fibrosis in mice (14). However, the majority of these studies were conducted in the context of dietary obesity, leaving a critical gap in understanding whether CIH alone can induce hepatic steatosis.

The human gut hosts an intricate bacterial community closely linked to various host physiologies (15, 16). The gut-liver axis is a consequence of a close anatomical and functional, bidirectional interaction of the gastrointestinal tract and liver, primarily through a portal circulation (17). It is well known that gut microbiota has an impact on hepatic disease via microbiota-derived metabolites and gut barrier dysfunction (17). Microbial-derived metabolites, particularly bile acids, serve as critical signaling molecules that regulate lipid metabolism, inflammation, and energy homeostasis (18, 19). In our previous review, we summarized that OSA could change the gut microbiota composition and then might negatively affect the cognitive, hepatic, and cardiovascular function (20). However, the specific microbial taxa and metabolites associated with CIH-related hepatic steatosis, particularly in the absence of dietary confounders, remain largely unexplored.

To address these gaps, we investigated the effects of CIH on hepatic steatosis in lean mice. Using an integrated multi-omics approach, we sought to characterize alterations in gut microbiota composition, circulating metabolites, and hepatic transcriptional programs in relation to CIH-induced hepatic steatosis. Furthermore, antibiotic intervention was employed to assess the contribution of gut microbiota to CIH-associated hepatic phenotypes. Our findings suggest that CIH exposure is associated with coordinated, phenotype-aligned changes in gut microbiota, bile acid profiles, and hepatic lipid-related pathways, including alterations involving *Bacteroides uniformis* and deoxycholic acid (DCA). These insights not only expand our understanding of OSA-related hepatic steatosis beyond obese populations but also identify potential microbiome-based therapeutic targets for clinical intervention.

## MATERIALS AND METHODS

### Patient characteristics

This study was conducted as part of the Shanghai Sleep Health Study and in accordance with the Declaration of Helsinki. All participants went to the hospital to seek medical advice because of snoring and provided written informed consent. The inclusion criteria were as follows: (i) participants aged 18 years or older; (ii) those who had undergone standard polysomnography (PSG). Exclusion criteria were (i) age younger than 18 years; (ii) previous treatment of OSA, including upper airway surgery, continuous positive airway pressure therapy, or oral appliance use; (iii) presence of severe comorbidities, including liver diseases, renal failure, and malignant tumors.

A total of 400 participants meeting the inclusion criteria were enrolled in this study. PSG was performed overnight and scored by trained technologists according to the 2012 American Academy of Sleep Medicine (AASM) criteria (21). The apnea-hypopnea index (AHI) was calculated as the total number of apneas and hypopneas per hour of sleep. For subgroup analysis, participants were further stratified by body mass index (BMI) into lean (BMI < 25 kg/m², *n* = 52) and overweight/obese (BMI ≥ 25 kg/m², *n* = 348) categories.

Fasting venous blood was collected the morning after PSG (approximately 7:00 a.m.–8:00 a.m.), and alanine aminotransferase (ALT) and aspartate aminotransferase (AST) were measured using routine clinical chemistry assays in the hospital laboratory.

### Hepatic steatosis assessment

Hepatic steatosis was assessed using the hepatic steatosis index (HSI), calculated according to the validated formula: HSI = 8 × (ALT/AST) + BMI + 2 (if type 2 diabetes present) + 2 (if female).

As BMI, sex, and diabetes status were integral components of the HSI formula, these variables were not included as covariates in regressions with HSI to avoid overadjustment. We employed a stratified analytical approach using a BMI cutoff of 25 kg/m² to examine the relationship between AHI and HSI. The primary analyses comprised two models: Model A evaluated the unadjusted Pearson association between AHI and HSI, and Model B evaluated the association with adjustment for age.

### Mice

All animal protocols were approved by the Animal Care Committee of Shanghai Sixth People’s Hospital affiliated to Shanghai Jiao Tong University School of Medicine. Male C57BL/6J mice (6–8 weeks old, 20–22 g body weight) were purchased from SPF Biotechnology Company (Beijing, China) and housed in a specific pathogen-free facility under controlled environmental conditions: 12-h light/dark cycle (lights on at 07:00), temperature 21°C ± 1°C, and relative humidity 50% ± 10%.

Following a 2-week acclimatization period with *ad libitum* access to standard chow diet (XTI01WC, Synergy Bio, China) and sterile water, mice were randomly assigned to experimental groups and group-housed (4 or 5 animals per cage). Food intake was measured on a per-cage basis by weighing the remaining chow once per week, and intake was normalized to the number of mice per cage and the number of days between measurements. Cage allocation was balanced across groups, and cages were treated as the experimental unit for food intake analyses.

In the 8-week CIH exposure study, each group consisted of 12 or 13 mice, while in the 16-week CIH exposure study, each group consisted of 9 or 10 mice. Animals that died prior to terminal collection were excluded from downstream assays. Not all assays were performed in all animals due to limited sample availability and pre-specified allocation. Animals included in each assay were selected prior to assay execution based on sample availability and quality control, without knowledge of outcome measures. Exact sample sizes for each assay were reported in the corresponding figure legends.

For terminal procedures, mice were deeply anesthetized with pentobarbital sodium prior to sample collection. Blood was collected by retro-orbital bleeding under deep anesthesia, followed immediately by cervical dislocation for euthanasia. Blood and tissue samples were collected during the same terminal procedure.

### CIH model

CIH was administered using the Oxycycler model A84 system (BioSpherix, Redfield, NY, USA) within a sealed chamber equipped with an integrated oxygen analyzer to continuously monitor and control oxygen concentration. Mice were exposed to CIH during the light phase for 8 h per day (09:00–17:00). Each hypoxic cycle consisted of a gradual reduction of the oxygen fraction from 21% to 5% ± 1% over a 150-s period, followed by rapid reoxygenation to normoxia (21% O₂) over the subsequent 150 s, resulting in a total cycle duration of 5 min (12 cycles per hour). Previous studies have shown that murine CIH paradigms with a nadir of 5%–6% O₂ induce intermittent hypoxia with arterial oxygen saturation levels comparable to those observed in patients with moderate-to-severe OSA (22, 23). Control mice were housed in identical chambers with continuous normoxia (21% O₂) for the same duration (13).

### Antibiotic treatment

To deplete the gut microbiota, mice received a broad-spectrum antibiotic cocktail by oral gavage every other day throughout the CIH exposure period. The antibiotic regimen included vancomycin (Sigma-Aldrich, Cat#V2002, 100 mg/kg/day), neomycin (Sigma-Aldrich, Cat#N6386, 200 mg/kg/day), metronidazole (Sigma-Aldrich, Cat#M3761, 200 mg/kg/day), and ampicillin (Sigma-Aldrich, Cat#A9518, 200 mg/kg/day), as previously described (24). Antibiotic administration was initiated concurrently with CIH exposure and continued for the entire 16-week experimental period. Control and CIH-only groups received vehicle gavage (sterile water) on the same schedule.

### Metabolic assessment

Body weight was measured weekly at the same time of day (09:00–10:00 a.m.). Following a 12-h overnight fast (water provided *ad libitum*), fasting blood glucose levels were measured via tail vein puncture using an Accu-Chek glucose monitor (Roche Diagnostics, Switzerland).

After a 12-h fasting, baseline glucose levels were measured. Mice then received an intraperitoneal injection of glucose solution (2.0 g/kg body weight; 20% [wt/vol] solution in sterile saline). Blood glucose concentrations were determined at 15-, 30-, 60-, 90-, and 120-min post-injection. Area under the curve (AUC) was calculated to assess glucose tolerance.

Upon completion of the CIH exposure protocol, mice were fasted for 12-h overnight and subjected to terminal sampling in the morning (09:00–11:00 a.m.). Under deep pentobarbital anesthesia, blood was collected by retro-orbital bleeding into EDTA-coated tubes and centrifuged at 3,000 × *g* for 10 min at 4°C to obtain plasma. Plasma samples were aliquoted and stored at −80°C until analysis, avoiding repeated freeze-thaw cycles. Plasma levels of ALT, AST, and triglycerides (TG) were measured using an automated biochemical analyzer (Chemray 240, Rayto Life and Analytical Sciences Co., Ltd., China). Following blood collection, mice were euthanized immediately by cervical dislocation, and tissues were harvested without delay.

### Histological analysis

For hepatic lipid assessment, fresh liver tissues were embedded in OCT compound (Sakura Finetek, Torrance, USA) and sectioned at 8 μm using a cryostat. Sections were fixed in 10% formaldehyde for 10 min, equilibrated in 60% isopropanol for 5 min, and stained with Oil Red O working solution for 10 min. Excess stain was removed by sequential rinsing with 60% isopropanol and distilled water. Oil Red O-positive area was quantified using ImageJ software. Three non-overlapping fields per section (20× magnification) were randomly selected from pericentral and periportal regions. The percentage of Oil Red O-positive area was calculated as (red pixels/total pixels) × 100%.

For morphological analysis, liver tissues were fixed in 10% formaldehyde at 4°C for 24 h and embedded in paraffin. Sections (5 μm) were deparaffinized in xylene, rehydrated through graded ethanol (100%, 95%, 80%, 70%), and stained with hematoxylin and eosin (H&E). Stained sections were dehydrated, mounted with neutral balsam, and examined by light microscopy.

### Transcriptome

Total RNA was extracted from mouse liver tissue, and 1 µg of high-quality RNA was used for mRNA sequencing library construction using the Vazyme mRNA sequencing library preparation kit (Vazyme, China). The quality of the prepared sequencing library was assessed using the Agilent 2100 Bioanalyzer (Agilent Technologies, USA). Subsequently, 2 ×150 bp paired-end sequencing was performed on the Illumina Novaseq 6000 sequencing platform (LC-Bio Technology CO., China). After quality control of raw data using Fastp software (https://github.com/OpenGene/fastp), the sequencing data were aligned to genome using HISAT2 (https://ccb.jhu.edu/software/hisat2). Genes or transcripts were assembled using StringTie software (https://ccb.jhu.edu/software/hisat2) and quantified by FPKM.

### Quantitative proteomics array

Hepatic inflammatory factors were quantified using a Mouse Th17 Array (RayBiotech, USA). Liver tissue samples stored at −80°C were thawed and homogenized, and total protein concentrations were determined by BCA assay. Prior to array analysis, all liver lysates were adjusted to the same total protein concentration (500 μg/mL), and an equal volume (100 μL) of each sample was loaded per well according to the manufacturer’s instructions.

The array consisted of a glass slide with 16 wells spotted in quadruplicate with capture antibodies against 18 mouse cytokines (IL-1β, IL-2, IL-4, IL-5, IL-6, IL-10, IL-12p70, IL-13, IL-17A, IL-17F, IL-21, IL-22, IL-23p19, IL-28A, IFN-γ, MIP-3α, TGF-β, and TNF-α). Arrays were incubated with serial dilutions of cytokine standards and protein samples overnight at 4°C. Following washing, detection antibody was added for 1 h at room temperature, followed by Cy3-equivalent dye-conjugated streptavidin for another hour. Signal intensity was measured using an Axon GenePix laser scanner (555 nm excitation, 565 nm emission), and cytokine concentrations (pg/mL) were determined using Q-Analyzer software v8.10.4 under equal protein-loading conditions.

### Metagenomic analysis

Following CIH exposure, mice were euthanized, and fecal samples were collected from the proximal colon. Fecal samples were snap-frozen in liquid nitrogen immediately after collection and stored at −80°C until DNA extraction. DNA was extracted using the E.Z.N.A. Stool DNA Kit (D4015, Omega Bio-tek, USA) according to the manufacturer’s instructions (25). Sequencing libraries were constructed using the TruSeq Nano DNA LT Library Preparation Kit (FC-121-4001, Illumina) and sequenced on the Illumina NovaSeq 6000 platform. Taxonomic classification was performed using Kraken2 software v2.1.1. Key bacterial taxa discriminating between groups were identified using linear discriminant analysis effect size (LEfSe) with a linear discriminant analysis (LDA) score >3.0 and *P* <0.05 (Kruskal-Wallis test).

### Untargeted metabolomics

Plasma samples (100 μL) were mixed with 500 μL of acetonitrile and frozen at −20°C for 2 h. After centrifugation at 20,000 × *g* for 10 min, the supernatant was dried in a freeze dryer and reconstituted in 100 μL of acetonitrile. All samples were analyzed using a TripleTOF 5600 Plus high-resolution tandem mass spectrometer (SCIEX, Warrington, UK) in both positive and negative ion modes. Reversed-phase separation was performed using an ACQUITY UPLC BEH C18 column (100 mm × 2.1 mm, 1.8 μm, Waters, USA). Samples were analyzed in randomized order. A pooled quality-control sample was injected at regular intervals to monitor instrument stability. Raw data were processed using Compound Discoverer 3.1.0 (Thermo Fisher Scientific, USA).

### Statistics

Statistical analyses were performed using SPSS software version 25.0. For longitudinal body weight and food intake, two-way repeated-measures ANOVA was used with time and treatment as factors, including the time × treatment interaction. For two-group comparisons, an unpaired two-tailed *t*-test (or Mann-Whitney *U* test for non-normal data) was applied. For multi-group comparisons, one-way ANOVA (or Kruskal-Wallis test) was used as indicated in figure legends. Data are presented as mean ± SEM unless otherwise stated. Two-tailed *P*-values <0.05 were considered to indicate statistically significant differences unless otherwise stated.

## RESULTS

### AHI is positively associated with hepatic steatosis

**Fig 1:**
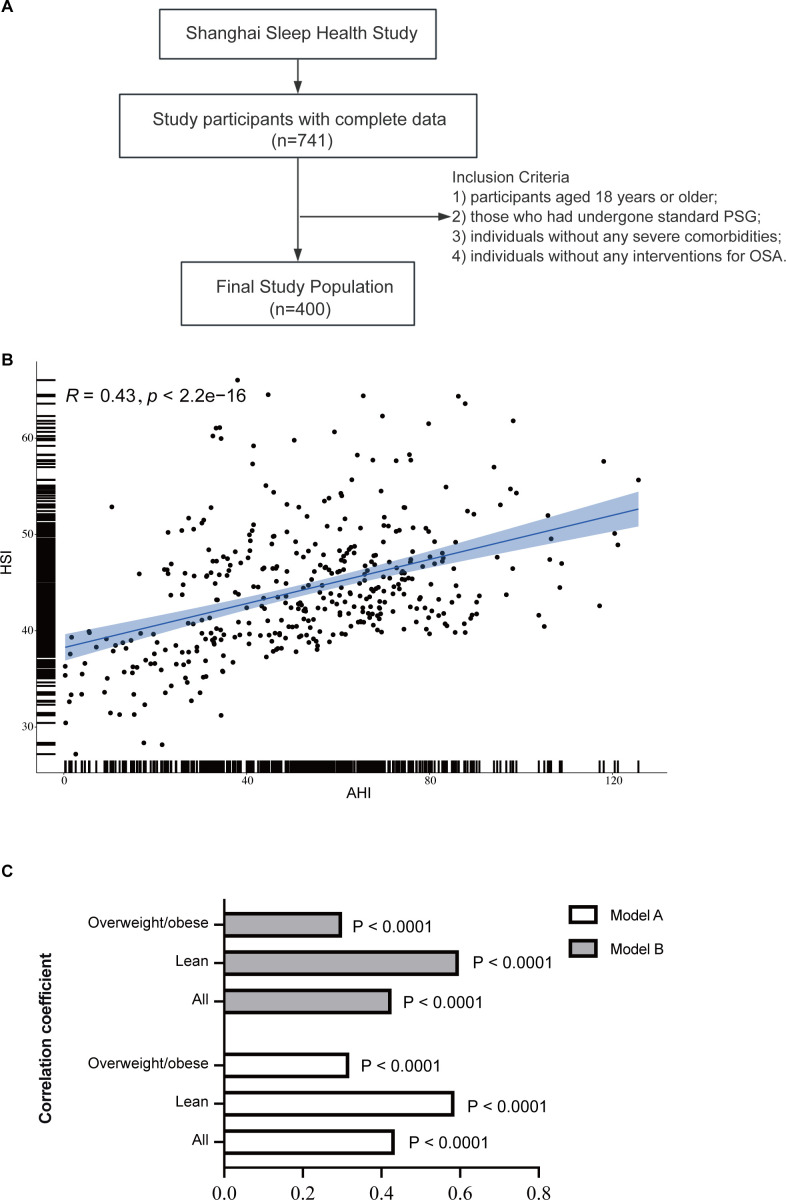
Observational association between apnea-hypopnea index (AHI) and hepatic steatosis index (HSI) in a clinical cohort. (**A**) Flowchart of participant selection from the Shanghai Sleep Health Study cohort. (**B**) Scatter plot showing the correlation between AHI and HSI after adjustment for age. The regression line represented the partial correlation between AHI and HSI, controlling for age. (**C**) Subgroup analysis comparing the association between AHI and HSI in lean (BMI < 25 kg/m², *n* = 52) versus overweight/obese (BMI ≥ 25 kg/m², *n* = 348) individuals. Model A evaluated the unadjusted Pearson correlation between AHI and HSI, while Model B evaluated the association with adjustment for age.

To investigate the relationship between OSA and hepatic steatosis, we examined the correlation between AHI and HSI in 400 participants from the Shanghai Sleep Health Study cohort, including both OSA patients and healthy controls (Fig. 1A). Our analysis revealed a significant positive correlation between AHI and HSI after adjusting for age (*R* = 0.43, *P* < 0.001; Fig. 1B), indicating that hypoxia events positively correlated with the degree of hepatic steatosis.

Further subgroup analysis was performed to explore this relationship across different BMI categories (lean: BMI < 25 kg/m², *n* = 52; overweight/obese: BMI ≥ 25 kg/m², *n* = 348). The analysis demonstrated that the positive association between AHI and HSI was consistent across both groups (lean: *R* = 0.586, *P* < 0.001; overweight/obese: *R* = 0.318, *P* < 0.001; Fig. 1C and Fig. S1), supporting an association between OSA severity and hepatic steatosis across BMI categories.

### Eight-week CIH exposure elicits early metabolic and transcriptional changes without obvious hepatic injury

**Fig 2:**
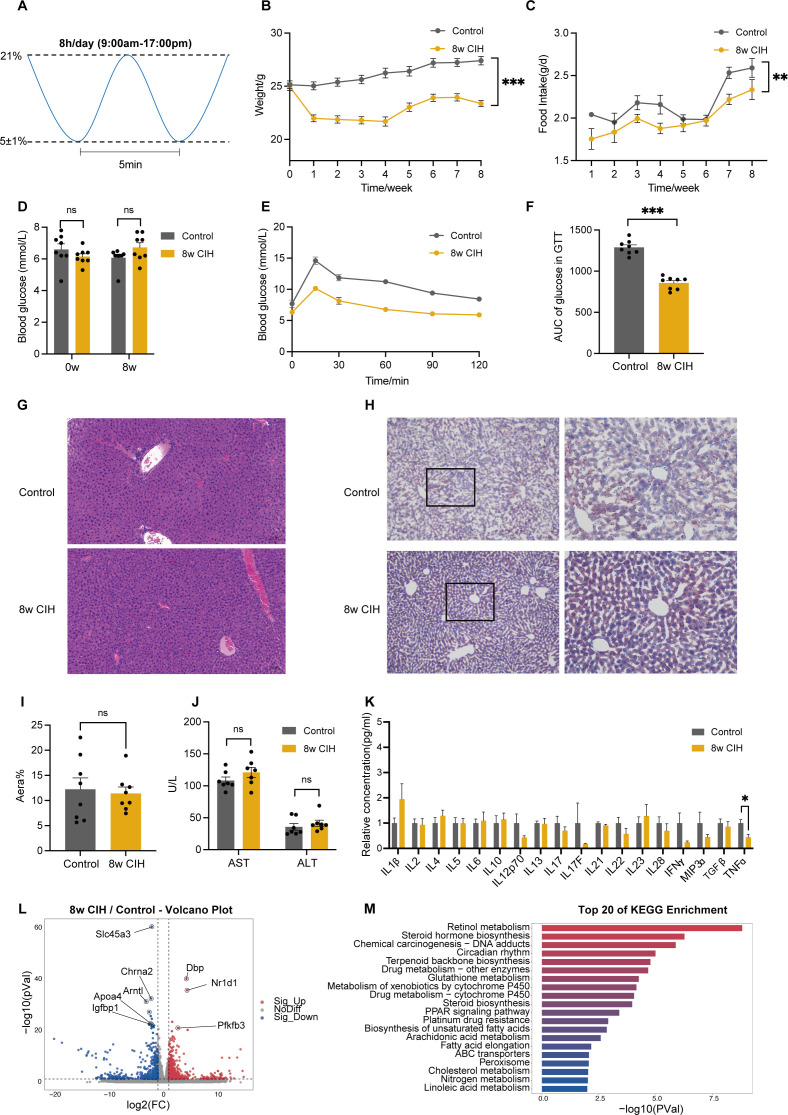
Eight-week CIH was not sufficient to induce the hepatic impairment in lean mice. (**A**) Pattern of IH exposure. (**B**) Body weight curves during the experimental period. Data were expressed as mean ± SEM (*n* = 12 or 13 per group). Statistical analysis was performed using two-way repeated-measures ANOVA, with time and treatment as factors. Significant main effects of time and treatment were observed, as well as a significant time × treatment interaction. The bracket and asterisks indicate the overall main effect of treatment across time. (**C**) Food intake. Food intake was measured weekly on a per-cage basis (three cages per group) and normalized to the number of mice per cage per day. Two-way repeated-measures ANOVA revealed significant main effects of time and treatment but no significant time × treatment interaction. The bracket and asterisks indicate the overall main effect of treatment across time. (**D**) Fasting blood glucose level. Data were expressed as mean ± SEM (*n* = 8 per group). Significance was calculated by unpaired *t*-test. (**E**) Blood glucose levels in response to glucose (2 g/kg body weight). Data were expressed as mean ± SEM (*n* = 8 per group). (**F**) Area under the curve (AUC) for glucose tolerance test. Significance was calculated by unpaired *t*-test. (**G**) Representative H&E staining of liver sections (original magnification, ×10). (**H**) Representative Oil Red O staining of liver sections (original magnification, ×20). (**I**) Oil Red O staining quantification. Data were expressed as mean ± SEM (*n* = 8 per group). Significance was calculated by unpaired *t*-test. (**J**) Plasma aspartate transferase (AST) and alanine transferase (ALT). Data were expressed as mean ± SEM (*n* = 7 per group). Significance was calculated by unpaired *t*-test. (**K**) Relative levels of 18 inflammatory factors in hepatic tissues. Data were expressed as mean ± SEM (*n* = 7 per group). Significance was calculated by unpaired *t*-test. (**L**) Volcano plot of differentially expressed genes in 8-week CIH versus control [*n* = 4 per group, |log_2_(fold change)| > 1, *P* < 0.05, Mann-Whitney *U* test]. Significance was calculated by Mann-Whitney *U* test. (**M**) KEGG pathway enrichment analysis of differentially expressed genes (top 20 pathways shown). ns, not statistically significant, ∗*P*< 0.05, ∗∗*P*< 0.01, ∗∗∗*P*< 0.001.

To further investigate how hypoxia events contribute to hepatic steatosis, a CIH model was performed to mimic the cyclical hypoxia-reoxygenation pattern in OSA. Eight-week-old male mice were maintained on a standard chow diet and exposed to intermittent hypoxia (5% O₂ at nadir, 12 cycles/h) or room air for 8 weeks (Fig. 2A). As expected, 8 weeks of CIH exposure significantly reduced body weight gain in lean mice (Fig. 2B). CIH-exposed mice exhibited a significantly lower overall level of food intake, while the temporal pattern of intake over time was comparable to controls (Fig. 2C).

We next assessed the impact of 8-week CIH exposure on glucose metabolism. Fasting blood glucose levels were similar between 8-week CIH-exposed mice and controls (Fig. 2D). Glucose tolerance test (GTT) showed lower glucose excursions in 8-week CIH-exposed mice compared with controls (Fig. 2E and F), suggesting preserved or potentially enhanced glucose handling at this early stage of CIH exposure in lean mice.

Liver histology assessed by H&E and Oil Red O staining showed no significant differences between 8-week CIH-exposed mice and controls (Fig. 2G, H and I). Consistently, levels of AST and ALT were comparable between the two groups (Fig. 2G through I). To evaluate hepatic inflammation, we measured 18 cytokines in hepatic tissues using a Mouse Th17 Array. Among the cytokines investigated, 17 showed similar levels between 8-week CIH-exposed mice and controls, with only TNF-α showing an obvious decrease in 8-week CIH group (Fig. 2K).

To gain deeper insights into the molecular changes induced by CIH, we performed transcriptome analysis of hepatic tissues. Differential gene expression analysis revealed upregulation of Dbp and Nr1d1, indicating disturbance of circadian rhythm caused by CIH exposure (Fig. 2L). In addition, upregulation of Pfkfb3 and downregulation of Arntl and Igfbp1 were observed, consistent with transcriptional changes related to hypoxia-responsive and HIF-1α-associated metabolic pathways. KEGG pathway enrichment analysis revealed significant enrichment of pathways related to retinol metabolism, steroid hormone biosynthesis, and circadian rhythm (Fig. 2M).

Therefore, these findings indicate that 8-week CIH exposure is associated with early metabolic and transcriptional responses in lean mice, occurring in the absence of obvious hepatic steatosis or liver injury.

### Sixteen-week CIH exposure is associated with hepatic inflammation and steatosis in lean mice

**Fig 3:**
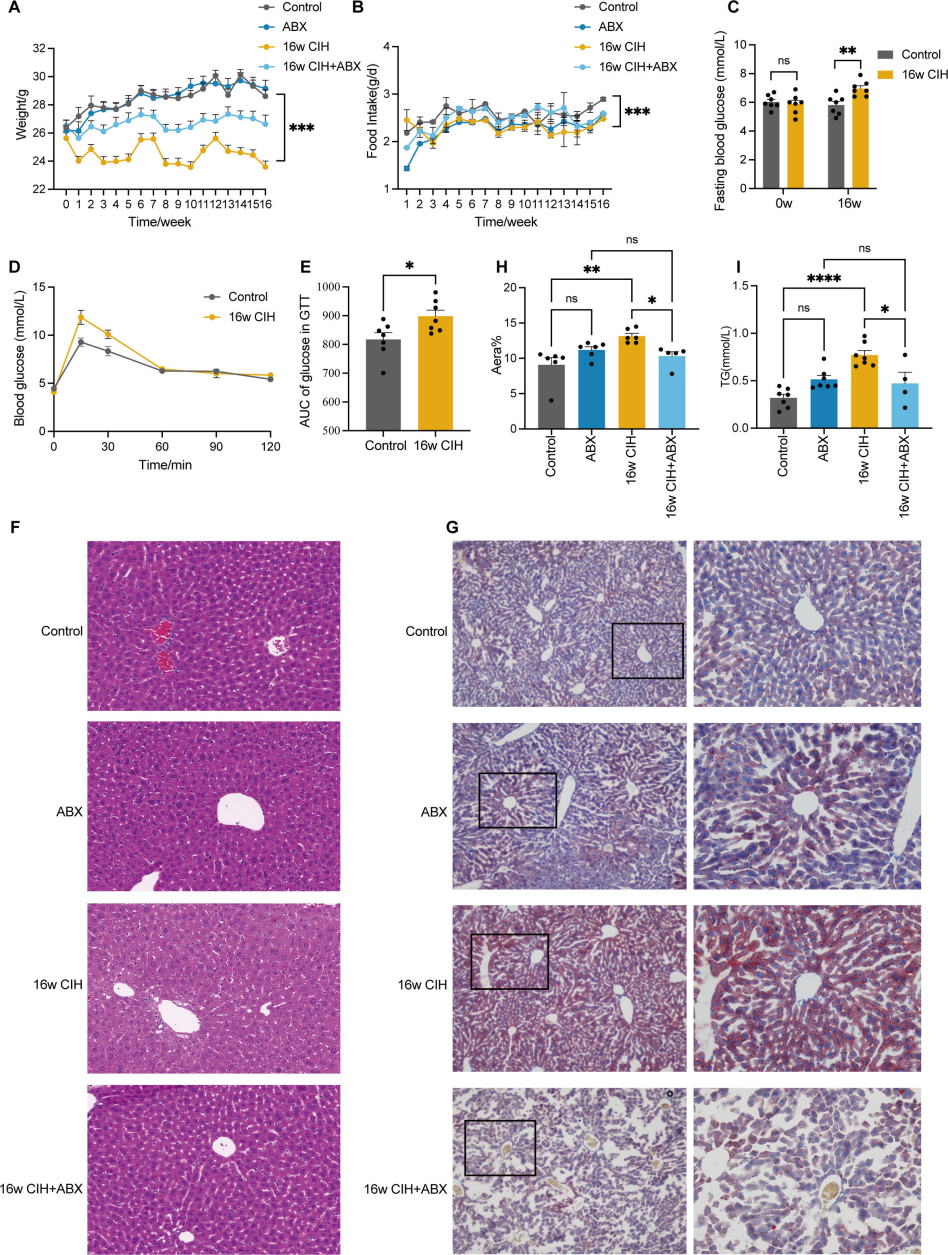
Sixteen-week CIH induced metabolic dysfunction and hepatic steatosis in lean mice. (**A**) Body weight curves during the experimental period. Data were expressed as mean ± SEM (*n* = 9 or 10 per group). Statistical analysis was performed using two-way repeated-measures ANOVA with time and treatment as factors. Significant main effects of time and treatment, as well as a significant time × treatment interaction, were observed. The bracket and asterisks indicate the overall main effect of treatment across time. (**B**) Food intake. Food intake was measured weekly on a per-cage basis (two cages per group) and normalized to the number of mice per cage per day. Data are expressed as mean ± SEM. Two-way repeated-measures ANOVA revealed significant main effects of time and treatment, whereas the time × treatment interaction was not significant. The bracket and asterisks indicate the overall main effect of treatment across time. (**C**) Fasting blood glucose level. Data were expressed as mean ± SEM (*n* = 7 per group). Significance was calculated by unpaired *t*-test. (**D**) Blood glucose levels in response to glucose (2 g/kg body weight). Data were expressed as mean ± SEM (*n* = 7 per group). (**E**) Area under the curve (AUC) for glucose tolerance test. Data were expressed as mean ± SEM (*n* = 7 per group). Significance was calculated by unpaired *t*-test. (**F**) Representative H&E staining of liver sections (original magnification, ×10). (**G**) Representative Oil Red O staining of liver sections (original magnification, ×20). (**H**) Oil Red O staining quantification. Data were expressed as mean ± SEM (*n* = 5 or 6 per group). Significance was calculated by one-way ANOVA. (**I**) Plasma triglyceride (TG) levels after 12-h fasting. Data were expressed as mean ± SEM (*n* = 4–7 per group). Significance was calculated by one-way ANOVA. ns, not statistically significant, ∗*P* < 0.05, ∗∗*P* < 0.01, ∗∗∗*P* < 0.001, ∗∗∗∗*P* < 0.0001.

To determine whether prolonged CIH exposure could induce hepatic impairment, we extended the exposure duration to 16 weeks. Throughout the experimental period, body weight and food intake were monitored (Fig. 3A and B). Sixteen-week CIH exposure resulted in metabolic dysfunction, including significantly elevated fasting glucose levels (control: 5.8 ± 0.7 mmol/L vs CIH: 7.0 ± 0.5 mmol/L, *P* < 0.01; Fig. 3C), and markedly impaired glucose tolerance (Fig. 3D and E), indicating that prolonged CIH exposure is accompanied by glucose metabolic abnormalities in lean mice.

Histological examination revealed marked hepatic changes following 16-week CIH exposure (Fig. 3F through H). H&E staining demonstrated increased microvesicular steatosis in liver sections from CIH-exposed mice (Fig. 3F). Oil Red O staining confirmed a significant increase in hepatic lipid content in CIH-exposed mice (Fig. 3G and H). Consistently, plasma TG levels were significantly elevated in 16-week CIH-exposed mice (Fig. 3I), further supporting the development of hepatic steatosis.

**Fig 4:**
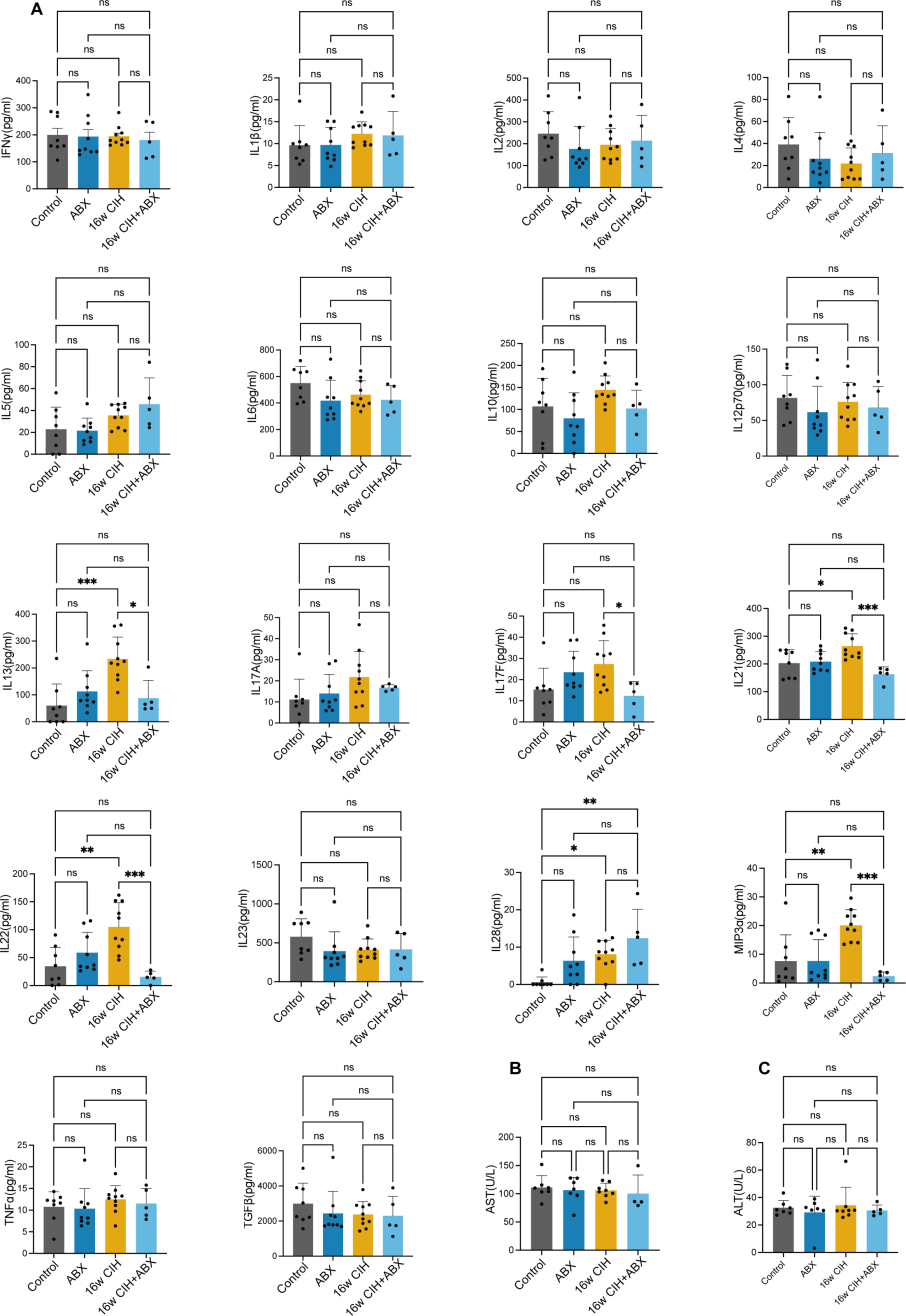
Sixteen-week CIH induced hepatic inflammation in lean mice. (**A**) Relative levels of 18 inflammatory factors in hepatic tissues. Data were expressed as mean ± SEM (*n* = 5–10 per group). Cytokine levels were normalized to total liver protein content. Significance was calculated by the Kruskal-Wallis test. (**B**) Plasma aspartate aminotransferase (AST). Data were expressed as mean ± SEM (*n* = 4–8 per group). Significance was calculated by Kruskal-Wallis test. (**C**) Plasma alanine transferase (ALT). Data were expressed as mean ± SEM (*n* = 5–8 per group). Significance was calculated by Kruskal-Wallis test. ns, not statistically significant, ∗*P* < 0.05, ∗∗*P* < 0.01, ∗∗∗*P* < 0.001.

Among the cytokines investigated, IL-13, IL-21, IL-22, IL-28A, and MIP-3α were significantly increased after 16-week CIH exposure (Fig. 4A), indicating an inflammatory response in the liver. However, plasma levels of AST and ALT remained comparable between groups (Fig. 4B and C), suggesting that hepatocyte damage had not yet occurred after 16-week CIH exposure.

Therefore, these findings demonstrate that 16-week CIH exposure is sufficient to induce hepatic steatosis and inflammatory responses in lean mice.

### Gut microbiota correlates with IH-induced hepatic steatosis

Increasing evidence has indicated that the gut microbiota and their metabolites directly influence hepatic lipid metabolism (26). In order to assess the contribution of gut microbiota in CIH-induced hepatic impairment, gut bacteria were depleted by administration of an antibiotic cocktail (ABX). Administration of ABX in the 16-week CIH group effectively alleviated the phenotype of hepatic steatosis, exhibiting decreased levels of TG in plasma and a decrease in micro-steatosis shown by H&E and Oil Red O staining (Fig. 3F through I). The addition of ABX in 16-week CIH was also significantly decreased the hepatic inflammation, especially in the levels of IL13, IL17F, IL21, IL22, and MIP3α (Fig. 4A). Moreover, administration of ABX would not influence the levels of AST and ALT (Fig. 4B and C). Together, the data indicate that gut microbiota might contribute to hepatic steatosis observed after prolonged CIH exposure.

### Gut microbiota composition is significantly altered after 16-week CIH exposure

**Fig 5:**
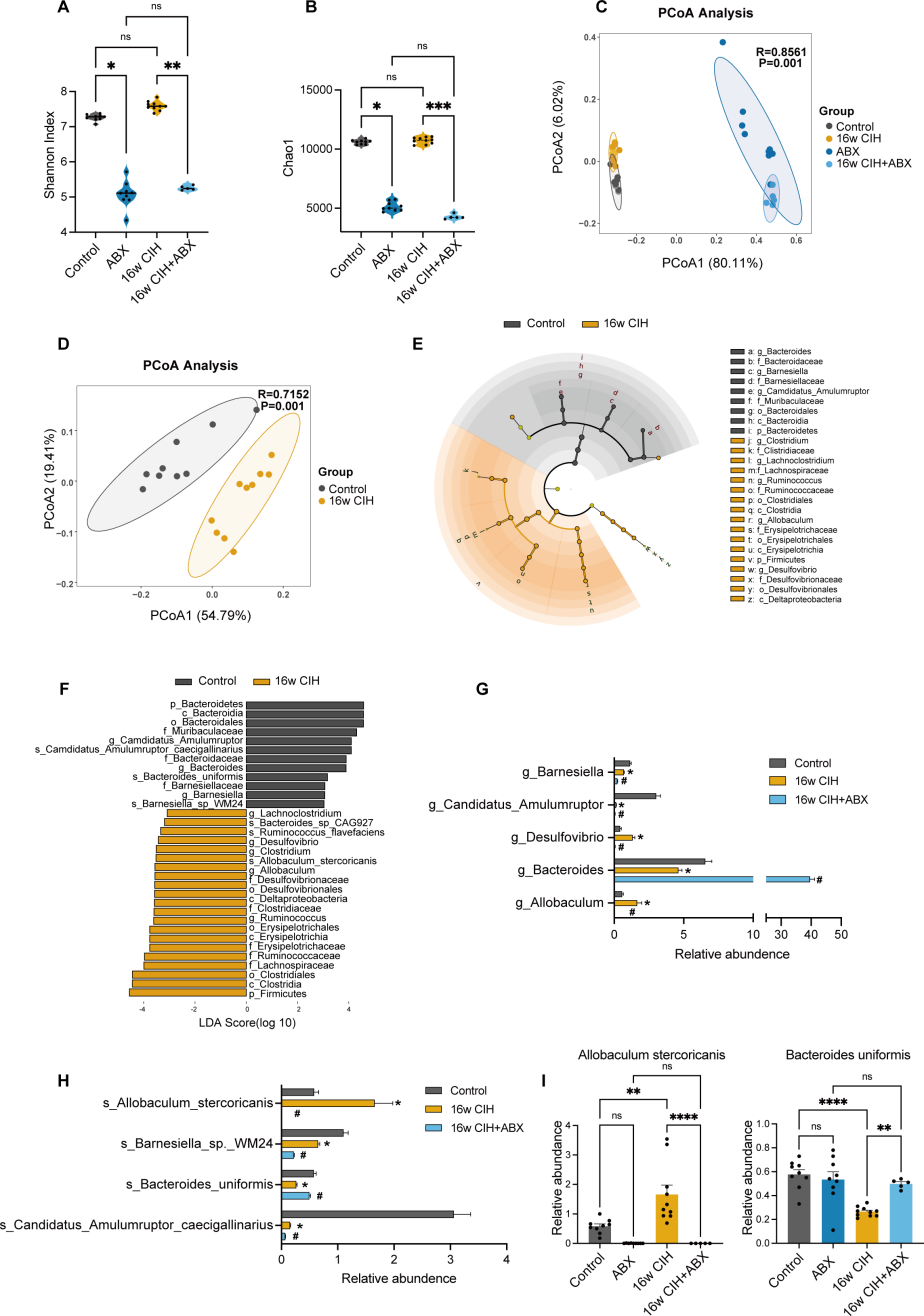
Alterations of gut microbial composition in mice following 16-week CIH exposure and antibiotic treatment (control *n* = 9, 16wCIH *n* = 10, ABX *n* = 9, 16wCIH + ABX *n* = 5). (**A, B**) Comparison of microbial α-diversity among four groups assessed by (**A**) Shannon index and (**B**) Chao1 index. Significance was calculated by the Kruskal-Wallis test. (**C**) Principal coordinate analysis (PCoA) based on Bray-Curtis dissimilarity showing β-diversity differences among all four groups. Significance was calculated by PERMANOVA. (**D**) PCoA based on Bray-Curtis dissimilarity comparing β-diversity between control and 16-week CIH group. Significance was calculated by PERMANOVA. (**E, F**) Linear discriminant analysis effect size (LEfSe) analysis identifying differentially abundant taxa between 16-week CIH and control groups (LDA score >3, *P* < 0.05). (**E**) Cladogram representation showing taxonomic hierarchy of enriched taxa. (**F**) LDA score bar plot of significantly different taxa. (**G, H**) Bacterial taxa whose abundances were altered by 16-week CIH and modulated by antibiotic treatment. * indicates taxa significantly changed in 16-week CIH vs control (adjusted *P* < 0.05); # indicates taxa significantly altered in 16-week CIH + ABX vs 16-week CIH (adjusted *P* < 0.05). Significance was calculated by the Kruskal-Wallis test with Benjamini-Hochberg adjustment. Data were expressed as mean ± SEM. (**G**) Bar plots show the relative abundance of selected genera. (**H**) Bar plots show the relative abundance of selected species. (**I**) Relative abundance of *Bacteroides uniformis* and *Allobaculum stercoricanis*. Data were expressed as mean ± SEM. Significance was calculated by one-way ANOVA. ∗*P* < 0.05, ∗∗*P* < 0.01, ∗∗∗*P* < 0.001, ∗∗∗∗*P* < 0.0001.

To investigate the impact of 16-week CIH on gut microbiota, we performed metagenomic sequencing on fecal samples from control, ABX, 16-week CIH, and 16-week CIH+ABX group. Alpha diversity analysis revealed while 16-week CIH exposure alone did not significantly affect microbial diversity, antibiotic treatment effectively depleted the gut microbiota as expected (Fig. 5A and B). Beta diversity analysis demonstrated distinct microbial community structures among these four groups, with clear separation between 16-week CIH and control groups (Fig. 5C and D).

LEfSe analysis identified multiple differentially abundant taxa between 16-week CIH and control group (Fig. 5E and F). At the genus level, the control group was enriched with *Bacteroides* and *Candidatus_Amulumruptor*, while the 16-week CIH group showed higher abundance of *Ruminococcus*. At the species level, *Bacteroides_sp._CAG:927, Prevotella_sp._MGM2, Ruminococcus_flavefaciens,* and *Allobaculum_stercoricanis* were significantly enriched in the 16-week CIH group, whereas *Candidatus_Amulumruptor_caecigallinarius, Barnesiella_sp._WM24,* and *Bacteroides_uniformis* species were more abundant in the control group.

To identify gut microbiota-dependent taxa that might be involved in 16-week CIH-associated effects, we applied the following filtering criteria: (i) differential taxa identified by LEfSe with a linear LDA score > 3.0 and *P* < 0.05; (ii) taxa significantly altered by 16-week CIH compared with the control group (adjusted *P* < 0.05); 3) taxa significantly altered by 16-week CIH + ABX compared with the 16-week CIH group (adjusted *P* < 0.05). We found that *Bacteroides*, *Desulfovibrio*, and *Allobaculum* were significantly altered by 16-week CIH and reversed by antibiotic treatment (Fig. 5G). At the species level, only *Bacteroides uniformis* and *Allobaculum stercoricanis* were significantly altered by 16-week CIH and reversed by antibiotic treatment (Fig. 5H). However, the abundance of *Allobaculum stercoricanis* was significantly decreased in the ABX group compared with the control, which did not correspond to the observed phenotype (Fig. 5I). Therefore, *Bacteroides uniformis* was selected for subsequent focused analyses, as its abundance was significantly decreased under 16-week CIH but not markedly changed among the control, ABX, or 16-week CIH + ABX groups.

### Gut microbiota-dependent metabolites might link CIH exposure to hepatic lipid accumulation

**Fig 6:**
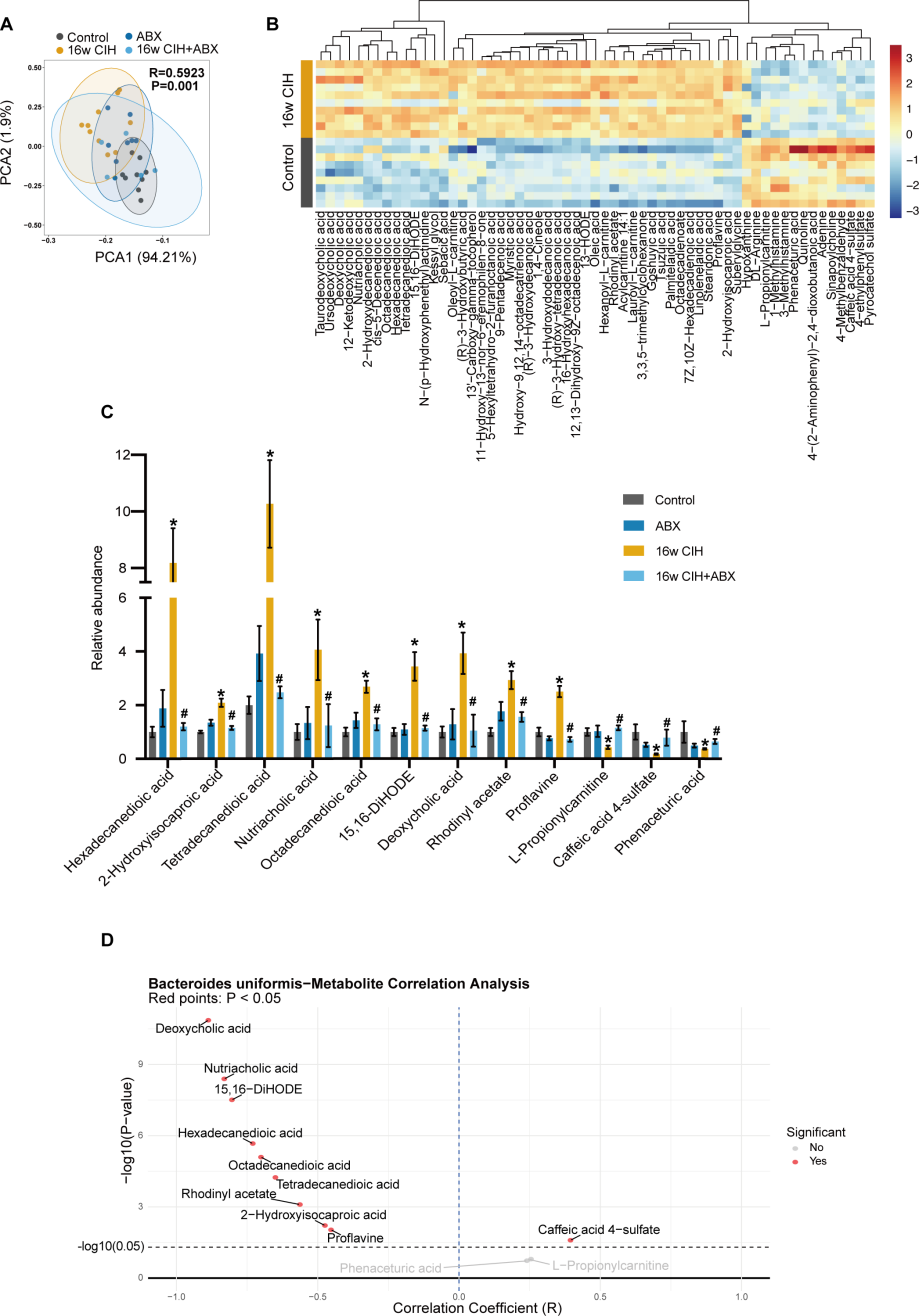
Alterations of plasma metabolites in mice following 16-week CIH exposure and antibiotic treatment (control *n* = 9, 16wCIH *n* = 10, ABX *n* = 8, 16wCIH + ABX *n* = 5). (**A**) Principal component analysis (PCA) of plasma metabolites showing distinct metabolic profiles. (**B**) Heatmap displaying differentially abundant metabolites between 16-week CIH and control groups. Metabolite abundance is shown as log2-transformed *z*-scores. Significantly altered metabolites were identified using Mann-Whitney *U* test with Benjamini-Hochberg adjustment (adjusted *P* < 0.05), VIP > 1, and |log_2_(fold change)| ≥ 1. (**C**) Bar plots showing relative abundance of gut microbiota-dependent metabolites. Data were presented as mean ± SEM. * indicates metabolites significantly changed in 16-week CIH vs control (|log₂FC| ≥ 1, VIP > 1, adjusted *P* < 0.05); # indicates taxa significantly altered in 16-week CIH + ABX vs 16-week CIH (|log₂FC| ≥ 1, VIP > 1, adjusted *P* < 0.05). (**D**) Spearman correlation between the selected microbiota-dependent metabolites levels and the abundance *of Bacteroides uniformis*. Red points indicate significant correlations (*P* < 0.05).

To elucidate the metabolic alterations underlying CIH-induced hepatic steatosis, we performed untargeted metabolomics analysis on plasma samples. PCA analysis revealed distinct metabolic profiles among groups, with clear separation between 16-week CIH-exposed and control mice (Fig. 6A). Numerous differentially abundant metabolites between 16-week CIH and control groups were identified (|log2FC| ≥ 1, VIP > 1, adjusted *P* < 0.05; Fig. 6B).

To identify microbiota-dependent metabolites mediating CIH effects, we applied the following filtering criteria: (i) metabolites significantly altered by 16-week CIH compared with the control group (|log₂FC| ≥ 1, VIP > 1, adjusted *P* < 0.05); (ii) metabolites significantly reversed by 16-week CIH + ABX compared with the 16-week CIH group (|log₂FC| ≥ 1, VIP > 1, adjusted *P* < 0.05); (iii) no significant difference between control and ABX. Twelve metabolites were identified after selection (Fig. 6C). Spearman correlation analysis was performed to determine metabolites closely associated with the abundance of *Bacteroides uniformis*. Ten metabolites showed significant correlations, among which DCA exhibited the strongest association (Spearman’s ρ = −0.89, *P* < 0.001; Fig. 6D). These results indicated that the abundance of *Bacteroides uniformis* was closely associated with bile acid metabolism, particularly DCA levels, under CIH conditions, suggesting a potential association between *Bacteroides uniformis* abundance, DCA levels, and hepatic lipid accumulation under CIH exposure.

### Transcriptomic analysis links PPAR signaling alterations to CIH-associated hepatic steatosis in an antibiotic-sensitive manner

**Fig 7:**
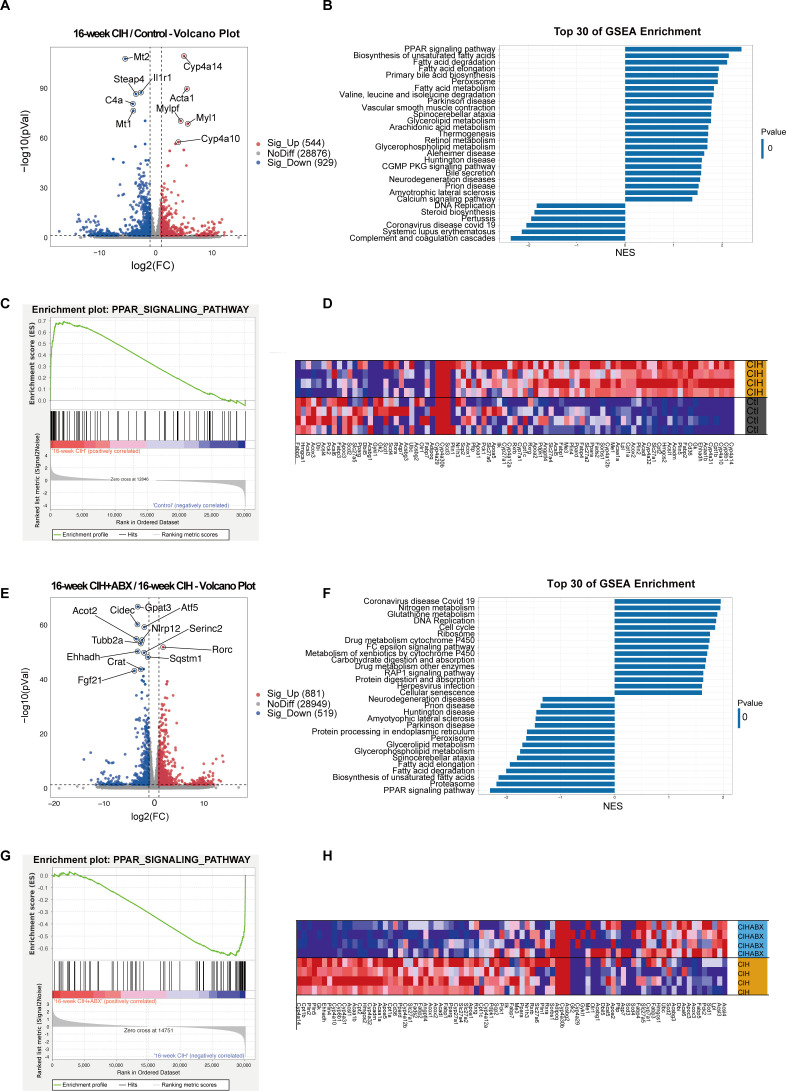
Transcriptomic analysis reveals CIH-associated, antibiotic-sensitive remodeling of PPAR signaling in the liver (*n* = 4 per group). (**A**) Volcano plot of the differentially expressed genes [*P* < 0.05 and |log2(fold change)| ≥ 1] of hepatic tissues in 16-week CIH group, compared with those in controls. Significance was calculated by Mann-Whitney *U* test. (**B**) GSEA analysis of enriched gene sets in the comparison of 16-week CIH mice from control mice. (**C**) The GSEA plot of the PPAR pathway (enrichment score [ES] = 0.70, normalized enrichment score [NES] = 2.40; nominal - value < 0.001) (**D**) The heatmap of differentially expressed genes related to the PPAR pathway in 16-week CIH group and control group (red: upregulated genes; blue: downregulated genes). (**E**) Volcano plot of the differentially expressed genes [*P* < 0.05 and |log2(fold change)| ≥ 1] of hepatic tissues in 16-week CIH + ABX group, compared with those in 16-week CIH group. Significance was calculated by Mann-Whitney *U* test. (**F**) GSEA analysis of enriched gene sets in the comparison of 16-week CIH + ABX mice from 16-week CIH mice. (**G**) The GSEA plot of the PPAR pathway (ES = −0.66, NES = −2.30; nominal *P*-value < 0.001). (**H**) The heatmap of differentially expressed genes related to the PPAR pathway in 16-week CIH + ABX group and 16-week CIH group (red: upregulated genes; blue: downregulated genes).

To further investigate the potential mechanism behind CIH-induced hepatic steatosis, transcriptome analysis of hepatic tissues was performed. A total of 544 upregulated genes and 929 downregulated genes were differentially expressed in the hepatic tissues of 16-week CIH/control group [*P* < 0.05 and |log2(fold change)| ≥ 1] (Fig. 7A). We conducted a gene set enrichment analysis (GSEA) of KEGG pathways to determine the pathways involved in the overall genetic change. KEGG pathway enrichment analysis showed significant activation of signaling pathways involved in PPAR pathway and fatty acid-related pathways (Fig. 7B and C). Expression levels of leading-edge genes related to the PPAR pathway were significantly increased in the 16-week CIH group, such as Cyp4a14, Cyp8b1, Cyp4a10, CD36, and Cpt1b (Fig. 7D).

To investigate the potential mechanism by which ABX improved hepatic lipid accumulation caused by 16-week CIH, transcriptome analysis of liver tissues in 16-week CIH + ABX group and 16-week CIH group was performed. A total of 881 upregulated genes and 919 downregulated genes were differentially expressed in the hepatic tissues of 16-week CIH + ABX/16-week CIH group [*P* < 0.05 and |log2(fold change)| ≥ 1], of which Srebf1 regulating the synthesis of cholesterol and fatty acids was significantly increased in 16-week CIH + ABX group (Fig. 7E). GSEA analysis showed significant inhibition of signaling pathways related to PPAR pathway (Fig. 7F and G). The genes decreased in the 16-week CIH + ABX group compared with the 16-week CIH group involved in the fatty acid metabolic process, and PPAR signaling pathway was coincidentally opposite to the 16-week CIH/control (Fig. 7B, D, F, and H). Transcriptome analysis demonstrated that dysregulation of genes involved in fatty acid metabolism and PPAR signaling might be involved in 16-week CIH-induced hepatic steatosis, with ABX treatment reversing these alterations.

## DISCUSSION

This study demonstrates that prolonged CIH induced hepatic steatosis and inflammatory changes in lean mice without concurrent obesity, highlighting a potential obesity-independent pathway linking OSA-related hypoxic stress to NAFLD. By integrating microbiome, metabolomic, and transcriptomic analyses, we identified coordinated alterations in gut microbial composition, bile acid profiles, and hepatic lipid-related transcriptional programs associated with CIH-induced hepatic steatosis. In addition, depletion of *Bacteroides uniformis* and elevation of DCA emerged as prominent, phenotype-aligned features that were sensitive to antibiotic intervention, alongside coordinated alterations in hepatic PPAR signaling. The present study does not establish a unidirectional causal pathway from a single microbial species to hepatic steatosis but rather identifies a coordinated microbiota-metabolite signature associated with CIH-induced hepatic pathology. While the precise causal relationships within this gut-liver axis require further mechanistic validation, our findings provide a framework for understanding how CIH-associated microbial and metabolic alterations may relate to hepatic steatosis in lean conditions. This represents an important step toward understanding hepatic steatosis pathogenesis in non-obese OSA patients.

Increasing evidence has indicated that patients with OSA had higher risk of NAFLD and NASH (12–14). Previous studies have mainly explored the role of CIH in high-fat-diet mice (13), leaving the effects of CIH under normal dietary conditions incompletely defined. Moreover, it has been reported that OSA severity is an independent risk factor for NAFLD even in non-obese populations (27). Therefore, our findings extend existing knowledge by showing that CIH alone is sufficient to promote the development of hepatic steatosis in lean mice and that modulation of the gut microbiota can substantially attenuate this phenotype. The observation that antibiotic treatment alleviated hepatic steatosis and inflammation under prolonged CIH exposure supports a contributory role of gut microbiota in this process.

Using metagenomic sequencing, we observed a consistent reduction in *Bacteroides uniformis* abundance following 16-week CIH exposure, accompanied by increased circulating DCA levels. Although alterations in *Bacteroides uniformis* and DCA have not been systematically examined in OSA patients or CIH-based animal models, emerging studies suggest a protective role of *Bacteroides uniformis* in hepatic lipid homeostasis and immune regulation (28–30). The abundance of *Bacteroides uniformis* and its associated bile acids has been reported in metabolic disease models, including type two diabetes mellitus mice (29). Restoration of *Bacteroides uniformis* improved lipid metabolism by regulating IRE1α-XBP1s axis (30) and TGR5/AMPK signaling pathway (29).

Although *Bacteroides uniformis* has been implicated in bile acid metabolic regulation (29, 31), a direct relationship between *Bacteroides uniformis* and DCA cannot be established based on the present data. Gut microbiota play a central role in shaping the bile acid pool by converting primary bile acids into secondary species, thereby increasing bile acid diversity and hydrophobicity (32). In turn, bile acids act as potent ecological regulators of the gut microbiota, influencing bacterial abundance and metabolic activity (33). Secondary bile acids, including DCA, possess detergent-like properties and can impose selective pressure on microbial populations through induction of cellular stress responses (33, 34). The observed elevation of circulating DCA under CIH conditions may reflect broader shifts in bile acid metabolism involving multiple microbial taxa and host regulatory pathways, rather than the effect of a single bacterial species. Conversely, elevated DCA may also act to suppress bile acid-sensitive taxa, including *Bacteroides* species, thereby reinforcing microbial compositional changes (34). Consistent with our observations, a cross-sectional study from Guatemala has shown that individuals with NAFLD had significantly higher circulating levels of DCA and its conjugate taurodeoxycholic acid (35). Excessive DCA has been implicated in NAFLD progression through bile acid-mediated signaling disturbances and microbiota-liver interactions (36). Further targeted mechanistic studies will be required to disentangle microbiota-bile acid feedback loops under CIH conditions.

The temporal patterns observed in this study suggest a transition from early compensatory responses to sustained pathological remodeling under prolonged hypoxic stress. While 8-week CIH was associated with metabolic and transcriptional changes in the absence of obvious hepatic steatosis or inflammation, 16-week CIH caused clear hepatic lipid accumulation and inflammation in lean mice, suggesting the existence of an early intervention window in OSA. Our 16-week CIH model more accurately reflected the long-term hepatic consequences of chronic OSA exposure. Previous studies have shown that short-term IH can exert beneficial effects, such as increasing erythropoietin levels (37), and improving cerebral blood flow (38). These findings indicate that while acute IH may trigger adaptive responses, chronic IH exposure leads to pathological hepatic impairment, highlighting the critical importance of early recognition and management of OSA to prevent long-term hepatic consequences.

Consistent with previous reports (39), CIH exposure in this study was associated with reductions in food intake and changes in body weight, raising the possibility that altered energy balance may contribute to CIH-associated hepatic phenotypes. We acknowledge that energy expenditure, substrate utilization, and lipid absorption were not directly assessed, and therefore the relative contribution of altered energy homeostasis cannot be fully excluded. CIH has been reported to influence systemic energy homeostasis and metabolic regulation, potentially contributing to hepatic lipid accumulation even in the context of reduced caloric intake (40). Future studies incorporating indirect calorimetry, pair-feeding designs, and lipid flux measurements will be required to more precisely delineate the roles of hypoxic stress, energy balance, and microbiota-dependent mechanisms in CIH-induced hepatic steatosis.

Our transcriptome analysis revealed alterations in PPAR signaling associated with CIH exposure, which were partially reversed by antibiotic treatment. CIH is known to increase metabolic stress on hepatocytes through hypoxia-related alterations in mitochondrial function and redox balance, which can secondarily engage PPAR-regulated transcriptional programs (10, 41, 42). Notably, many PPAR target genes involved in fatty acid uptake and oxidation, including Cyp4a, Cd36, and Cpt1b, were upregulated under CIH conditions in this study. However, the upregulation of these genes does not necessarily indicate PPAR pathway overall dysfunction. Instead, it may reflect a compensatory response, whereby the liver attempts to increase fatty acid oxidation to cope with lipid accumulation under CIH stress.

Although elevated DCA levels coincided with PPAR-related transcriptional changes in this model, the present data do not establish a direct causal link between DCA and PPAR activation. Bile acids predominantly exert their biological effects through specific receptors, such as FXR and TGR5 (43), and any crosstalk between bile acid signaling and PPAR pathways is generally indirect and context dependent. In addition, expression levels of canonical bile acid receptors, including FXR and TGR5, were not significantly altered in hepatic tissue from CIH-exposed mice compared with controls. While changes in receptor activation cannot be inferred from mRNA expression alone (43), these data do not provide evidence for functional activation of classical bile acid receptor pathways in this model. Therefore, the observed modulation of PPAR signaling may reflect downstream or parallel metabolic and inflammatory responses to CIH. The partial normalization of PPAR-related gene expression following antibiotic treatment further supports a microbiota-dependent component of CIH-associated metabolic remodeling. Together, these findings suggest that altered PPAR signaling reflects an adaptive or compensatory transcriptional response to CIH-induced metabolic stress, rather than a primary driver of hepatic pathology.

Our findings have important clinical implications. First, OSA patients should be screened for NAFLD regardless of BMI, as CIH independently induces hepatic steatosis. Second, *Bacteroides uniformis* abundance and DCA levels may represent potential non-invasive biomarkers reflecting CIH-associated metabolic disturbances, although clinical validation is required. Moreover, microbiota-targeted or bile acid-modulating strategies warrant further investigation as potential adjunctive approaches for OSA-associated liver disease. However, several limitations of this study should be acknowledged. First, the antibiotic intervention was designed to perturb the gut microbial community at the ecosystem level rather than to influence a single bacterial species (24, 44). Accordingly, although antibiotic treatment attenuated CIH-associated hepatic steatosis and inflammation, these findings support a microbiota-dependent component of the observed phenotype rather than establishing definitive causal roles for individual taxa such as *Bacteroides uniformis*. Definitive species-level causality will require targeted approaches, including bacterial supplementation, fecal microbiota transplantation, or germ-free model experiments. In addition, it should be noted that broad-spectrum antibiotic treatment can exert systemic effects independent of gut microbiota depletion, including direct influences on host mitochondrial function and inflammatory status (45). Therefore, the attenuation of hepatic steatosis observed following antibiotic treatment should not be interpreted as evidence of a purely microbiota-mediated mechanism, but rather as reflecting antibiotic-sensitive processes that may involve both microbiota-dependent and microbiota-independent components. Second, although our transcriptomic analyses identified coordinated alterations in PPAR signaling associated with CIH exposure and antibiotic sensitivity, these data reflect changes at the mRNA level and do not define direct regulatory mechanisms. Further mechanistic studies will be required to clarify the precise role of PPAR signaling in CIH-associated hepatic metabolic remodeling. Finally, the translation of these findings to human OSA populations will require longitudinal clinical studies incorporating integrated microbiome and metabolomic profiling.

In conclusion, this study reveals that 16-week CIH exposure induces hepatic steatosis in lean mice and is associated with coordinated, antibiotic-sensitive alterations in gut microbiota composition, bile acid metabolism, and hepatic lipid-related transcriptional programs. Notably, depletion of *Bacteroides uniformis*, accumulation of DCA, and concomitant changes in PPAR signaling emerged as phenotype-aligned features under CIH exposure. These findings provide an obesity-independent framework for understanding OSA-associated hepatic steatosis and highlight gut microbiota-host metabolic interactions as an important area for future mechanistic and translational research.

## Data Availability

The transcriptome data have been deposited in the Genome Sequence Archive (GSA) at National Genomics Data Center (NGDC) under accession number CRA038869. The metagenomic sequencing data generated in this study have been deposited in OMIX at NGDC under accession number OMIX015154. The plasma metabolomics data set has been deposited in OMIX at NGDC under accession number OMIX015151.

## SUPPLEMENTAL MATERIAL

Figure S1 msystems.00163-26-s0001.docx BMI-stratified correlation analysis between AHI and HSI.

ASM does not own the copyrights to Supplemental Material that may be linked to, or accessed through, an article. The authors have granted ASM a non-exclusive, world-wide license to publish the Supplemental Material files. Please contact the corresponding author directly for reuse.
