# Supplementary material for: Chronic intermittent hypoxia exacerbates hepatic steatosis in a microbiota-dependent manner in lean mice
Source: mSystems. 2026 May 4;11(6):e00163-26. doi: 10.1128/msystems.00163-26 (PMC13289086; doi:10.1128/msystems.00163-26)
Supplement: Figure S1 — BMI-stratified correlation analysis between AHI and HSI. [file msystems.00163-26-s0001.docx]

**Chronic intermittent hypoxia exacerbates hepatic steatosis in a microbiota-dependent manner in lean mice**

Xiaoman Zhang^1*^, MD, Anyuan Zhong^2*^, MD, Yupu Liu^1#^, MD, PhD, Jianyin Zou^1#^, MD, PhD, Meizhen Gu^3#^, MD, PhD, Xiaoyue Zhu^1#^, PhD, Huajun Xu^1^, MD, PhD, Shankai Yin^1^, MD, PhD


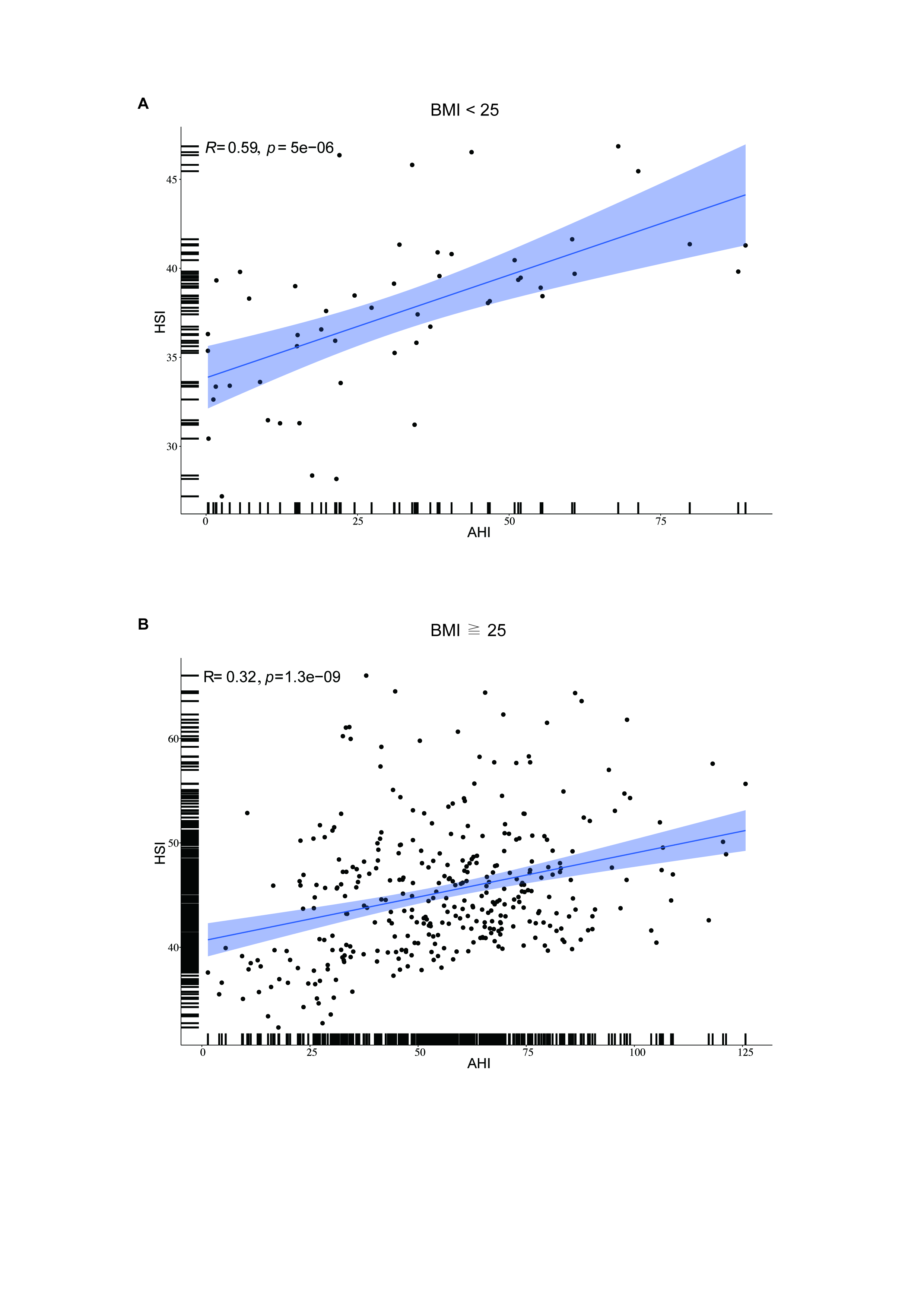


**Supplementary Figure 1.** BMI-stratified correlation analysis between AHI and HSI in (A) lean (BMI <25 kg/m²) and (B) overweight/obese (BMI ≥25 kg/m²) participants with adjustment for age. The regression line represented the partial correlation between AHI and HSI, controlling for age.
